# Supplementary figures and images for: Evaluation of underweight status may improve identification of the highest-risk patients during outpatient evaluation for pulmonary tuberculosis
Source: PLoS One. 2020 Dec 11;15(12):e0243542. doi: 10.1371/journal.pone.0243542 (PMC7732099; doi:10.1371/journal.pone.0243542)

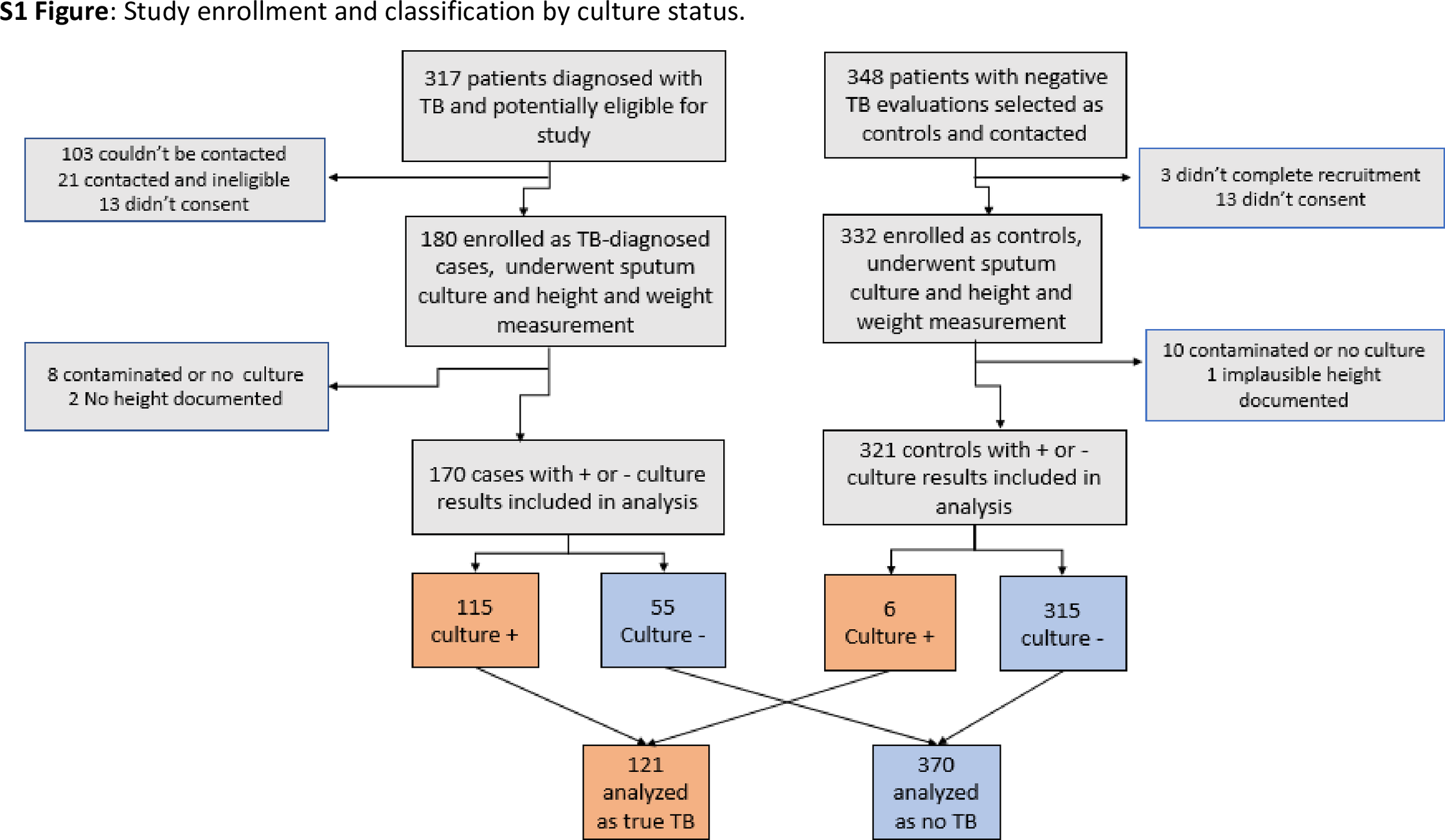

Supplement: S1 Fig — (TIF) [file pone.0243542.s001.tif]

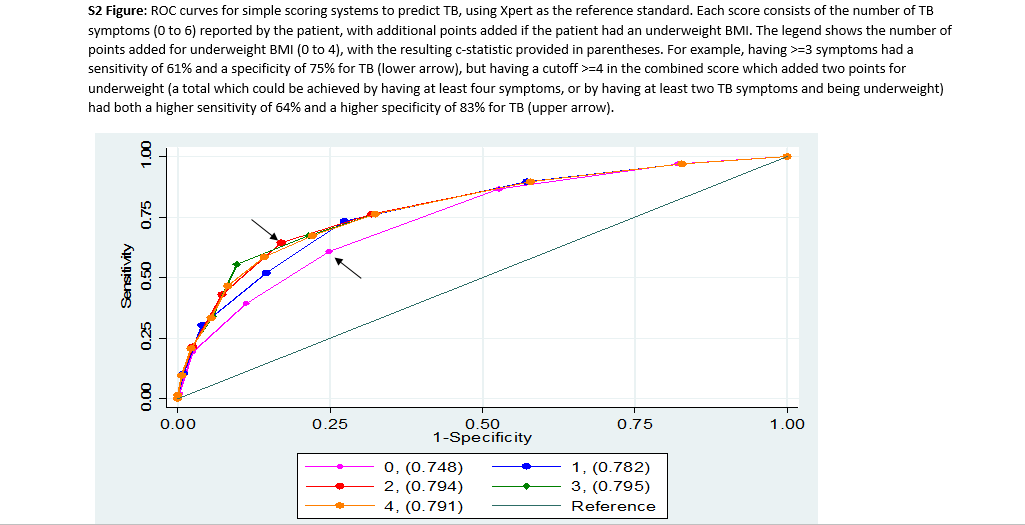

Supplement: S2 Fig — (TIF) [file pone.0243542.s002.tif]
