# Supplementary material for: Evaluation of underweight status may improve identification of the highest-risk patients during outpatient evaluation for pulmonary tuberculosis
Source: PLoS One. 2020 Dec 11;15(12):e0243542. doi: 10.1371/journal.pone.0243542 (PMC7732099; doi:10.1371/journal.pone.0243542)
Supplement: S1 Table — (DOCX) [file pone.0243542.s003.docx]

**S1 Table**: Accuracy of individual symptoms (comparing Normal and underweight status) in predicting Xpert-positive TB among adult presumptive at four clinics in Kampala, Uganda. (Prevalence=14%)

**Symptom Normal weight status (n = 271)**

|  | **n (%)** | | **Sensitivity**(95%CI) | | | **Specificity**(95%CI) | | | | **PPV**(95%CI) | | | **NPV**(95%CI) | | | |  |  |  |  |
| --- | --- | --- | --- | --- | --- | --- | --- | --- | --- | --- | --- | --- | --- | --- | --- | --- | --- | --- | --- | --- |
|  |  | |  | | | |  | | |  |  | | | | | |  |  |  |  |
| Chronic Cough | | 162 (60%) | | | 79%(67, 88) | 46%(39, 53) | | | 19%(17, 22) | | | 93%(89, 96) | | |  | | | |  |  |
|  | |  | | |  |  | | |  | | | | |  | |  | | |  |  |
| Fevers | | 75(28%) | | | 48%(35, 61) | 78%(72, 84) | | | 27%(20, 34) | | | 90%(88, 92) | | |  | | |  | | |
|  | |  | | |  |  | | |  | | |  | | |  | | |  | | |
| Night Sweats | | 58(21%) | | | 44%(32, 58) | 85%(80, 90) | | | 33%(25, 43) | | | 90%(88, 92) | | |  | | |  | | |
|  | |  | | |  |  | | |  | | |  | | |  | | |  | | |
| Weight Loss | | 119(44%) | | | 66%(52, 77) | 62%(56, 69) | | | 23%(18, 27) | | | 92%(88, 94) | | |  | | |  | | |
|  | |  | | |  |  | | |  | | |  | | |  | | |  | | |
| Chest pain | | 101(37%) | | | 54%(41, 67) | 68%(61, 74) | | | 22%(17, 27) | | | 90%(87, 92) | | |  | | |  | | |
|  | |  | | |  |  | | |  | | |  | | |  | | |  | | |
| Hemoptysis | | 10(4%) | | | 7%(2, 16) | 97%(94, 99) | | | 28%(10, 57) | | | 86%(85, 87) | | |  | | |  | | |
|  |  | | |  | | | |  | | |  | | | | | |  |  | |  |

**Symptom Underweight status (n = 109)**

|  | **n(%)** | | **Sensitivity**(95%CI) | | **Specificity**(95%CI) | | | | | **PPV**(95%CI) | | **NPV**(95%CI) | | | | | | |  | | | |  | |  |  |
| --- | --- | --- | --- | --- | --- | --- | --- | --- | --- | --- | --- | --- | --- | --- | --- | --- | --- | --- | --- | --- | --- | --- | --- | --- | --- | --- |
|  |  | |  | |  | | | | |  | |  | | | | | | |  |  |  |  |  |  |  |  |
| Chronic Cough | | 86(79%) | | 91%(81, 97) | |  | | 40%(25, 56) | | | 20%(16, 24) | | |  | | | 96%(92, 98) |  | | | |  |  |  |  |  |
|  | |  | |  | |  | | |  | |  | |  | | |  | | | |  |  |  |  |  |  |  |
| Fevers | | 44(40%) | | 46%(33, 58) | | 67%(52, 81) | | | | | 19%(12, 28) | |  | | | 88%(85, 91) | |  | | | | | | | |  |
|  | |  | |  | |  | | | | |  | |  | | |  | | | |  | | | | | |  |
| Night Sweats | | 42(39%) | | 47%(35, 60) | | 74%(56, 87) | | | | | 23%(15, 35) | |  | | | 89%(86, 92) | |  | | | | | | | |  |
|  | |  | |  | |  | | | | |  | |  | | |  | | | |  | | | | | |  |
| Weight Loss | | 82(75%) | | 86%(76, 94) | | 42%(27, 58) | | | | | 20%(16, 25) | |  | | | 95%(90, 97) | |  | | | | | | | |  |
|  | |  | |  | |  | | | | |  | |  | | |  | | | |  | | | | | |  |
| Chest pain | | 51(47%) | | 50%(37, 63) | | 58%(42, 73) | | | | | 17%(12, 23) | |  | | | 88%(83, 91) | |  | | | | | | | |  |
|  | |  | |  | |  | | | | |  | |  | | |  | | | |  | | | | | |  |
| Hemoptysis | | 5(5%) | | 8%(3, 17) | | 100%(92, 100) | | | | | 100% (-) | |  | | | 87%(86, 88) | |  | | | | | | | |  |
|  |  | |  | |  | | | | |  | |  | | | | | | |  | | | | |  | |  |
|  |  | |  | | | |  | | |  | | | | |  | | | |  | | | | |  | |  |

PPV: Positive Predictive Values NPV: Negative Predictive Value
