# Supplementary material for: Evaluation of underweight status may improve identification of the highest-risk patients during outpatient evaluation for pulmonary tuberculosis
Source: PLoS One. 2020 Dec 11;15(12):e0243542. doi: 10.1371/journal.pone.0243542 (PMC7732099; doi:10.1371/journal.pone.0243542)
Supplement: S2 Table — (DOCX) [file pone.0243542.s004.docx]

**S2 Table**: Accuracy of number of TB symptoms (using two simple scoring systems) in predicting Xpert-positive TB among adult presumptive patients at four clinics in Kampala, Uganda. (n = 465, Prevalence=14%)

**Cutoff**  No Additional Points for Underweight **2 Additional Points for Underweight**

|  | Sensitivity(95%CI) | Specificity(95%CI) | Sensitivity(95%CI) | Specificity(95%CI) |  |
| --- | --- | --- | --- | --- | --- |
| ( >=1 ) | 97%(93, 99) | 18%(14, 22) | 97%(93, 99) | 17%(13, 22) |  |
|  |  |  |  |  |  |
| ( >=2 ) | 87%(80, 92) | 47%(42, 53) | 90%(83, 94) | 42%(37, 48) |  |
|  |  |  |  |  |  |
| ( >=3 ) | 61%(52, 69) | 75%(70, 80) | 76%(68, 83) | 68%(63, 73) |  |
|  |  |  |  |  |  |
| ( >=4 ) | 39%(31, 48) | 89%(85, 92) | 64%(56, 73) | 83%(79, 87) |  |
|  |  |  |  |  |  |
| ( >=5 ) | 19%(13, 27) | 98%(95, 99) | 43%(35, 52) | 93%(89, 95) |  |
|  |  |  |  |  |  |
| ( >=6 ) | 2%(0.4, 6) | 100%(98, 100) | 22%(15, 29) | 98%(95, 99) |  |
|  |  |  |  |  |  |
| ( >=7 ) | N/A | N/A | 10%(5, 16) | 99%(98, 100) |  |
|  |  |  |  |  |  |
| ( >=8 ) | N/A | N/A | 2%(0.2, 5) | 100%(99, 100) |  |
|  |  |  |  |  |  |
|  |  |  |  |  |  |
