# Supplementary material for: Evaluation of underweight status may improve identification of the highest-risk patients during outpatient evaluation for pulmonary tuberculosis
Source: PLoS One. 2020 Dec 11;15(12):e0243542. doi: 10.1371/journal.pone.0243542 (PMC7732099; doi:10.1371/journal.pone.0243542)
Supplement: S3 Table — (DOCX) [file pone.0243542.s005.docx]

**S3 Table**: Positive predictive value (PPV) of symptom count plus two additional points for underweight status, using Xpert MTB/RIF as the reference standard for TB, at multiple cutoff values and multiple prevalences of TB. n=465

| **Prevalence** | 14% (study population) | | 5% | | 15% | | 30% | |
| --- | --- | --- | --- | --- | --- | --- | --- | --- |
| **Cut off** | PPV(95%CI) | | PPV(95%CI) | | PPV(95%CI) | | PPV(95%CI) | |
| ( >=1 ) | | 16%(16, 17) | | 6%(5, 6) | | 17%(16, 18) | | 34%(32, 35) |
|  | |  | |  | |  | |  |
| ( >=2 ) | | 21%(19, 22) | | 8%(7, 8) | | 22%(20, 23) | | 40%(37, 43) |
|  | |  | |  | |  | |  |
| ( >=3 ) | | 29%(25, 32) | | 11%(10, 13) | | 30%(26, 34) | | 51%(46, 55) |
|  | |  | |  | |  | |  |
| ( >=4 ) | | 39%(33, 45) | | 17%(13, 21) | | 40%(34, 47) | | 62%(55, 68) |
|  | |  | |  | |  | |  |
| ( >=5 ) | | 50%(39, 60) | | 24%(17, 32) | | 51%(50, 62) | | 72%(62, 80) |
|  | |  | |  | |  | |  |
| ( >=6 ) | | 60%(41, 76) | | 32%(18, 50) | | 61%(42, 77) | | 79%(64, 89) |
|  | |  | |  | |  | |  |
| ( >=7 ) | | 73%(38, 92) | | 46%(16, 79) | | 74%(39, 93) | | 87%(61, 97) |
|  | |  | |  | |  | |  |
| ( >=8 ) | | 100% | | 100% | | 100% | | 100% |
|  |  | |  | |  | |  | |
