# Supplementary material for: Evaluation of underweight status may improve identification of the highest-risk patients during outpatient evaluation for pulmonary tuberculosis
Source: PLoS One. 2020 Dec 11;15(12):e0243542. doi: 10.1371/journal.pone.0243542 (PMC7732099; doi:10.1371/journal.pone.0243542)
Supplement: S1 Instrument — (PDF) [file pone.0243542.s006.pdf]

S1 Instrument: Participant interview questions relevant to the current analysis
English Version
1.0 Record ID

____

1.1. Date of interview

____

1.2. Interviewer ID

____

2.0. Demographics
2.1. Record the sex of the participant
Male
Female

2.2. What is your age, in years?

____

3.0. Special types of data to be collected by study staff
3.1. Weight in kg

____

(Enter '-999' if not done/ unknown.

3.2. Height in meters

____

(Enter '-999' if not done/ unknown.

3.3. Do you currently smoke tobacco?
Yes
No
Refused to answer

3.4. Did you smoke tobacco (>100 cigarretes or it's equivalent) in the past?
Yes
No
Refused to answer

3.4. What is the highest grade of education that you have attained?
None
PLE Certificate
O-level certificate
A-level certificate
Degree
Any Tertiary education (non- University)
Any postgraduate education
Attained postgraduate degree
Unknown/refused

3.6. Which of the following best describes your occupation?
Self-employed
Student
Salaried worker
Housewife
Occasional work (piece jobs)
Unemployed but able to work
Unemployed and unable to work
Unknown/refused

3.7. Now I am going to ask you some questions about your use of alcoholic drinks during this past year (meaning the past 12 months). How often did you have a drink containing alcohol in the past year?
Never
Monthly or less
Two to four times a month
Two to three times per week
Four or more times a week
Refused

4.0. Symptom and care-seeking history

4.1. Which of the following symptoms do you have currently? (Currently can mean today or within the past few days.) Select all that apply.
Cough
Coughing up blood

Unexplained fever or chills
Drenching sweats at night
Shortness of breath
Pain in my chest
None of the above
Unknown/refused

4.1.1. You said that you currently have a cough. Now looking back in time, for how long have you had this cough? ________ (Round to the nearest week ; < 3 days= 0 weeks, 4-10 days = 1 Week, etc.) (Interviewer should record answer in weeks.)

4.1.2. You said that you currently have been Coughing up blood. Now looking back in time, for how long have you been Coughing up blood? ________ (Round to the nearest week ; < 3 days= 0 weeks, 4-10 days = 1 Week, etc.) (Interviewer should record answer in weeks.)

4.1.3. You said that you currently have a fever or chills. Now looking back in time, for how long have you had fever or chills? _______ (Round to the nearest week; < 3 days= 0 weeks, 4-10 days = 1 Week, etc.) (Interviewer should record answer in weeks.)

4.1.4. You said that you currently have sweats at night. Now looking back in time, for how long have you had sweats at night? ______ (Round to the nearest week; < 3 days= 0 weeks, 4-10 days = 1 Week, etc.)
(Interviewer should record answer in weeks.)

4.2. Within the past twelve months, have you experienced weight loss of more than 5kg, or enough to make your clothes loose?
Yes
No
Unknown/refused

Luganda Version

1.0. Ennamba eyawula ekiwandiiko

____

1.1. Ennaku z'omwezi z'okuddamu ebibuuzo

____

1.2. Ennamba eyawula abuuza ebibuuzo

____

2.0. Ebikwata ku muntu

2.1. Wandiika ekikula ky'omuntu eyenyigiramu

Musajja

Mukazi

2.2. Olina emyaka emeka? ___________________________________

3.0. Obubaka obw'ebika eby'enjawulo ebinakunganyizibwa abakozi mukunonyereza

3.1. Obuzito mu kg ___________________________________

(Yingiza '999' bwekiba tekikoleddwa/ tekimanyiddwa)

3.2. Obuwanvu mu metres(ng'okozesa olukoba olupima)

____

(Yingiza '999' bwekiba tekikoleddwa/ tekimanyiddwa)

3.3. Onywa taaba mukiseera kino?

Yee

Nedda

3.4. Wanywa kutaaba(eminwe >100 egya sigala) mukiseera ekiyise?

Yee

Nedda

3.5. Daala ki ely'okusoma elisinga okuba elya waggulu lyewali ofunye

Tewali
Satifikeeti oba ebbaluwa ya PLE
Satifikeeti oba ebbaluwa ya O-level
Satifikeeti oba ebbaluwa ya A-level
Diguli
Okusoma kwonna okugattibwa ku diguli esooka
Yafuna diguli ey'okubiri
Tekimanyiddwa/yagaanye

3.6. Kiliwa kubino wammanga ekisinga okunyonyola omulimo ggwo?
Yekozesa
Muyizi
Mukozi aweebwa omusaala
Mukyala akola egy'awaka
Emirimu egibeerawo olusi n'oluusi (emirimo emimpi)
Talina mulimo naye asobola okukola
Talina mulimo ate nga tasobola kukola
Tekimanyiddwa/yagaanye

3.7. Kati ngenda kukubuuza ebibuuzo ebimu ku nkozesayo eyebyokunywa ebitamiiza mu mwaka oguyise. Mirundi emeka gyewanywa eky'okunywa ekilimu ebitamiiza mu mwaka oguwedde?
Tewali murundi gwonna
Buli mwezi oba ekitono okusingawo
Emirundi ebbiri okutuuka ku ena omwezi
Eirundi ebbiri okutuuka ku essatu buli wiiki
Emirundi ena oba okusingawo mu wiiki
Yagaanye

4.0. Akabonero n'ebyafaayo by'okunoonya obujanjabi
4.1. Kaliwa kububonero bunno k'obadde nako mu wiiki eyise? Londa bwonna obujaawo.
Okukolola
Okukolola omusaayi

Omusujja ogutanyonyoddwa oba ekitengo
Okukoowa ennyo okutanyonyoddwa
Okutuuyana okuyitiridde ekiro
Okugwebwaako omukka mukusa
Obulumi mukifuba
Tewali kubyonna waggulu
Tekimanyiddwa/agaanye

4.1.1. Omaze baanga ki ng'olina ekifuba (wandiika ekidibwaamu mu wiiki)
____
(Genda ne wiiki esinga okuba okumpi; < ennaku 3 = wiiki 0, Ennaku 4-7 = Wiiki 1)

4.1.2. Omaze baanga ki ng'okolola omusaayi ?
____
(wandiika ekidibwaamu mu wiiki) (Genda ne wiiki esinga okuba okumpi; < ennaku 3 = wiiki 0, Ennaku 4-7 = Wiiki 1)

4.1.3. Omaze baanga ki ng'olina omusujja oba ekitengo?
____
(wandiika ekidibwaamu mu wiiki) (Genda ne wiiki esinga okuba okumpi; < ennaku 3 = wiiki 0, Ennaku 4-7 = Wiiki 1)

4.1.4. Omaze baanga ki ng'ofuna okutuuyana ekiro?
____
(wandiika ekidibwaamu mu wiiki) (Genda ne wiiki esinga okuba okumpi; < ennaku 3 = wiiki 0, Ennaku 4-7 = Wiiki 1)

4.2. Mu mwaka oguyise, oyise mu kukoga okusukka mu kilo 5, oba okumala okuleetera engoye zo okukulebera?
Yee
Nedda
Tekimanyiddwa/yagaanye
