# Supplementary material for: Evaluation of underweight status may improve identification of the highest-risk patients during outpatient evaluation for pulmonary tuberculosis
Source: PLoS One. 2020 Dec 11;15(12):e0243542. doi: 10.1371/journal.pone.0243542 (PMC7732099; doi:10.1371/journal.pone.0243542)
Supplement: Attachment — Submitted filename: Reviewers comments.doc [file pone.0243542.s007.doc]

Reviewer comments

Evaluation of underweight status may improve management among adults being evaluated for pulmonary tuberculosis in Kampala, Uganda.

**Abstract**

- Author should clearly define the main purpose of this study
- Line 32-33: Patients reported median two symptoms (interquartile range [IQR] 0, 6). The following sentence is not clear .

Materials & methods

- Could you please reorganize this section as follow?
  - Study setting & design;
  - study participants (population and sampling): based on fig 1 , please provide clearly explanation and you should complete the last step or line TB+ 121 & TB- 363.
  - study variables
  - data collection process
  - Statistical analysis
  - Ethical considerations (please provide the number for reference)
- Line 87-88: All individuals had their weight (in kg) and height (in meters) taken using a SECA-216 stadiometer and weighing scale (Seca Industries, Hamburg). *Please revise this sentence …. It is a bit confusing.*

Results

- Table 1 : Please provide the P value or mark by star the significant results and use footnotes

Conclusion:

Line 297-298: In summary, our results suggest that ***BMI assessment and number of symptoms should be included*** as part of the clinical decision-making process among adults presenting for evaluation of possible pulmonary TB.
