# Supplementary material for: Evaluation of underweight status may improve identification of the highest-risk patients during outpatient evaluation for pulmonary tuberculosis
Source: PLoS One. 2020 Dec 11;15(12):e0243542. doi: 10.1371/journal.pone.0243542 (PMC7732099; doi:10.1371/journal.pone.0243542)
Supplement: Attachment — Submitted filename: Response_to_Reviewers-[PONE-D-20-28238R].docx [file pone.0243542.s009.docx]

REVIEWERS COMMENTS

Reviewer #1:

Evaluation of underweight status may improve management among adults being evaluated for pulmonary tuberculosis in Kampala, Uganda.
Abstract
- Author should clearly define the main purpose of this study

**Response: Following the reviewer’s guidance, we added Lines 25 – 26 stating ‘**’We sought to evaluate the usefulness of BMI assessment and a count of characteristic TB symptoms for identifying patients at highest risk for TB.**’’**

- Line 32-33: Patients reported median two symptoms (interquartile range [IQR] 0, 6). The following sentence is not clear.

**Response: This has been edited to state, “**Of the six symptoms assessed, the median number of symptoms that patients reported was two (interquartile range [IQR]: 1, 3).**’’ Lines 36 - 37.**

Materials & methods
- Could you please reorganize this section as follow?
o Study setting & design;
o study participants (population and sampling): based on fig 1 , please provide clearly explanation and you should complete the last step or line TB+ 121 & TB- 363.
o study variables
o data collection process
o Statistical analysis
o Ethical considerations (please provide the number for reference)

**Response: We have followed the Reviewer’s suggestion as stated above to reorganize the sections. See Lines 80 - 181. We have also added more detail to S1 Figure, including enumeration of the initial population we attempted to recruit and the final numbers classified as having or not having culture positive TB.**

- Line 87-88: All individuals had their weight (in kg) and height (in meters) taken using a SECA-216 stadiometer and weighing scale (Seca Industries, Hamburg). Please revise this sentence …. It is a bit confusing.

**Response: This has been revised to read ‘**’Research staff measured all participants’ weight (in kg) and height (in meters) using a SECA-216 weighing scale and stadiometer (Seca Industries, Hamburg).**’’ Lines 111 -113**

Results
- Table 1: Please provide the P value or mark by star the significant results and use footnotes

**Response: We have revised Table 1 to indicate significant associations in bold; this is now explained in a footnote.**

Conclusion:
Line 297-298: In summary, our results suggest that BMI assessment and number of symptoms should be included as part of the clinical decision-making process among adults presenting for evaluation of possible pulmonary TB.

**Response:**

**Though we were unable to see the comment attached to this line in the manuscript, we have modified our conclusion in response to comments from Reviewer 2 as explained below, and we now make the more modest claim that “**identification of patients with underweight BMI adds discriminatory power for identifying patients with pulmonary TB, and that routine BMI assessment should therefore be considered in settings where clinicians are often required to make decisions about empiric TB treatment for high-risk patients while awaiting bacteriologic data**”. Lines 336 - 340**

Reviewer #2:

 i. Introduction:
Query 1: The author states, “Most discussions of clinical decision-making for TB center on risk factors for developing TB and on the presence and severity of characteristic symptoms. Body mass index (BMI) is often not given the same consideration but may be useful in identifying high-risk patients.”

Loss of weight is one of the main constitutional symptoms for tuberculosis (TB), and therefore this, in addition to any of the other constitutional symptoms remains a useful tool for clinical diagnosis of TB. Since weight is one of the components used to calculate the BMI, it is not surprising that low BMI will also be associated to clinical TB diagnosis. I therefore wonder if using BMI improves TB management as stated in the title or it contributes to confirming the clinical impression of TB?

**Response:**

**Our objective was to understand what additional information BMI would add after accounting for other TB symptoms – including recent weight loss. To clarify this for the reader, we added a phrase ‘’…** but it is not clear to what extent BMI assessment provides additional information independent of weight loss or other constitutional symptoms’’ (**Lines 67 - 68)**. **We further edited Line 72 to read,** ‘’Determination of BMI is inexpensive and could, if shown to be sufficiently valuable, be incorporated into routine triage,” **and Line 75 to state** ‘’… the degree of independence between TB symptoms and BMI is uncertain’’**.**

**We agree that weight loss and low BMI are related; however, we found that the correlation was weak, with 44% (125/284) of the participants who reported the symptom ‘weight loss’ falling within ‘normal weight BMI’ and 24% (28/118) of those with underweight BMI answering “no” when we asked about weight loss (Table 2: Line 229). These results, along with the association of underweight BMI with TB after adjusting for weight loss and other symptoms in our regression analysis (Table 1), suggest that BMI assessment provides additional value in identifying patients likely to have true TB, beyond what can be ascertained from a clinical interview.**

**To further clarify our objectives, we have modified our title to offer more precision, to focus on whether BMI ‘’may improve identification of the highest-risk patients….” rather than “improve management”. Lines 1-3**

As correctly stated, TB treatment centers particularly in low income countries struggle with carrying out basic clinical parameters such as weight and blood pressure, usually because of lack of availability of the necessary equipment. Thus an additional measure of height and then the calculation of BMI is very desirable but rather impractical. I am not sure about the relevance and translation of suggesting the use of BMI as an additional clinical tool for TB diagnosis.

**Response:** **The reviewer is correct that our study does not demonstrate feasibility of incorporating height and weight measurement or BMI calculation into routine clinical care, nor does it necessarily show that BMI calculation would provide additional value beyond a clinician’s impression that a patient appears underweight. We have added discussion of this (“**Whether this estimated improvement in classification is sufficiently meaningful to merit measuring BMI in every patient likely depends on the availability of equipment (e.g., stadiometers) and the additional burden imposed on clinic staff by conducting BMI assessment.**”, lines 310 - 313). Our study suggests a next step of studying the practicality of performing height, weight measurements and other tools that can be employed in especially resource-limited settings. Lines 319 – 323.**

ii. Methods:
Query 2: “We analyzed six symptoms: chronic cough (defined as cough lasting at least two weeks), fevers, weight loss (unexplained and sufficient to make clothes loose), chest pain, coughing up blood (hemoptysis), and night sweats (drenching).”
The authors measured six symptoms. I noted the omission of anorexia which is also one of the main constitutional symptoms of TB. Is there any reason why this was left out in the assessment?
What would add value is assessing a symptom or sign outside the main constitutional symptoms and evaluating this to aid in confirming clinical diagnosis of TB such as blood pressure or any other.
**Response: The symptoms that we chose to evaluate are WHO’s list of characteristic TB symptoms (which includes cough, fever, night sweats, and weight loss, but not anorexia; see references 1 & 2), as well as additional symptoms indicating severity of pulmonary symptoms (hemoptysis, chest pain, and duration of cough) given that all patients in our study population reported cough. We agree with the reviewer, however, that it would be useful for future studies to evaluate a broader range of constitutional symptoms and clinical findings, to understand which are correlated and which add additional predictive power.**

Query 3: “Body Mass Index (BMI) was computed as weight (kg) divided by height (meters squared) and categorized as underweight (less than 18.5 kg/m2), normal weight (18.5 to 24.9 kg/m2), overweight (25 to 29.9 kg/m2) or obese (over 30kg/m2).”

Was the BMI calculation carried out on site at the TB treatment centers? Who calculated the BMI? Knowledge of this will help in confirming feasibility. If the BMIs were calculated at the point of statistical analysis, the suggestion of using BMI in clinical care will then remain theoretical.

**Response: The purpose of our current analysis was to evaluate the potential utility of BMI assessment and not its practical application. At the TB treatment units, the weight (kg) and height (m) were measured only for participants (negative or positive on X-pert test, or clinically diagnosed) who consented to participate in the research study (after their diagnostic evaluation was completed). Our revised methods state that these measurements were taken by research study staff (Line 111-113). The BMI was then calculated at statistical analysis. It is therefore unknown the practicality of facility health worker performing the BMI, as we stated in response to Query 1 above. We have also noted in our revised limitations that “**clinical utility will depend in part on the feasibility of BMI assessment in a busy and resource-constrained clinical setting.”**(Line 321-322)**

Query 4: “A multivariable logistic regression model was used to explore the relationship between BMI, number of TB symptoms and TB status.”
The author needs to describe these models better stating what variables were adjusted, what levels of significance were considered. Was it stepwise forward or backward?

**Response: We have refined our approach and clarified it in our methods section. We selected a limited number of covariates for analysis, a priori, based on their known or hypothesized association with TB. In this revision, we performed logistic regression with backward selection from among these pre-identified covariates, at p >0.2; we also verified that forward selection selected the same covariates for inclusion in our final model. (Lines 148-150)**

Query 5: The study was approved by the Ethics Review Committee of the Makerere University School of Public Health, Kampala-Uganda.

Please add the ethical approval reference number. Was ethical approval sought to use data from the TB registries at the study sites?

**Response: Ethical approval was sought to use data from the TB registries. We have added the approval reference number. (Line 178-181)**

iii. Results:

Query 6: “Participants were patients aged ≥15 years who underwent diagnostic evaluations for possible pulmonary TB at four TB Diagnostic and Treatment Units in Kampala, Uganda (including one large public clinic, two smaller private clinics, and an HIV clinic). TB evaluation at these facilities typically included sputum Xpert MTB/RIF testing [Cepheid, Inc] according to routine clinical laboratory procedures. Between 22nd May 2018 and 29th February 2020, patients who received a TB diagnosis were invited to enroll in our study; except during specified months at the largest clinic (during which all patients were eligible regardless of location of residence), enrollment was limited to residents of certain nearby zones.”
“BMI, TB symptoms, and culture status were determined for 121 patients with culture-positive TB and 363 patients with negative culture results (S1 Fig).”

The study enrolled participants over a period of almost two years in a setting where there is bound to have a high number of TB patients. The authors have not stated how many TB patients they actually screened and enrolled. They immediately report only on the number that was evaluated for sputum culture. It is also not clear how they selected the patients for sputum culture? Were there any potential biases?

**Response: We have followed the Reviewer’s guidance. Under the methods section, we have included how our participants were selected from the total 665 potentially eligible participants between 22^nd^ May 2018 and 29^th^ February 2020. We have also updated S1 Figure (as a flow chart) to show how we reached the participants we included in our analysis. The revised S1 figure shows the patients who were eligible but not enrolled for various reasons, and indicates more clearly that sputum culture was attempted on all who were enrolled. Our methods also note that geographic criteria based on zone of residence were used to restrict eligibility in a relatively unbiased fashion (Lines 92-93)**

Were BMIs calculated on all the patients they enrolled or for only those that had a sputum culture result?
The results presented were all based on those with a TB culture result. They didn’t describe the rest of the population. It would have been good to have a general description of the whole study population and then after focus on those with a culture result.

**Response: Our revised methods also clarify that both culture and BMI were performed at the time of enrollment for all enrolled participants. (Lines 111-116)**

iv. Discussion:
Query 7: “A clearer understanding of the clinical signs and symptoms most predictive of TB could help improve the early management of patients presenting for diagnosis of pulmonary TB, particularly in situations where reliable diagnostic tests are unavailable or their results are likely to be delayed. In this study, we estimated the accuracy of number of TB symptoms alone and in combination with BMI in identifying culture-positive pulmonary TB among patients presenting to health facilities in Kampala, Uganda.”

I am not sure that the clinical signs and symptoms most predictive for TB were assessed independently in this study. This can be done since most of the constitutional symptoms of TB were collected with the exception of anorexia. This would further confirm if weight loss or low weight independently have strong associations with a positive TB culture. If they do, then if would be sufficient to rely on them without the additional impractical measurement of a BMI. However, calculation of BMI is still highly recommended for quality clinical care.
The authors could have also explored other symptoms or signs and assessed their prediction independently or in addition to the constitutional TB symptoms. This would have added more to the existing knowledge gap.

**Response: While our specific objective in this analysis was to understand what BMI assessment would add in the prediction of pulmonary tuberculosis, we appreciate the reviewer’s input on the importance of understanding the extent to which weight loss and other constitutional symptoms could serve as a proxy for BMI. Based on this feedback, our revised regression analysis adjusts for weight loss and other symptoms, and we also report results on the correlation between self-reported weight loss and underweight status. Our results indicate independent strong associations of both weight loss and low weight with positive TB culture. (Table 1, Line 199)**

Query 8: “Our findings support the inclusion of BMI among the clinical indicators that are considered by clinicians when they consider empiric initiation of TB treatment.”
I think this is a sweeping statement especially since we are not sure who and where the BMIs were calculated. And also because TB patients present with a history of weight loss, as well as actual low weights. Therefore, the BMI measurements of these patients are also bound to be low. I am not sure about the additional value of adding BMI measurement in already struggling health systems.

**Response: Following the guidance of the Reviewer, we have edited this statement to read, “**The c-statistic difference that we estimated suggests that inclusion of BMI in a risk assessment could increase (by approximately 4%) the probability that a patient with true TB would be selected for empiric treatment before one without TB, beyond what could be ascertained by assessing the patients’ pulmonary and constitutional symptoms alone.” **(Line 303-306).**

**We further add a phrase, ‘’**Whether this estimated improvement in classification is sufficiently meaningful to merit measuring BMI in every patient likely depends on the availability of equipment (e.g., stadiometers) and the additional burden imposed on clinic staff by conducting BMI assessment.**” (Lines 310 - 313)**

v. Conclusion:
Query 9: “Although the clinical utility of any specific algorithm would require further validation, these findings suggest that consideration of BMI adds diagnostic value above and beyond symptom count alone.”
This statement needs to be revised especially since there are methodological weakness with the choice of using BMI as main component, selection of patients assessed, and lack of clear regression model analysis description.

**Response: We have revised our summary paragraph to be more circumspect. We now state that, “**In summary, our results suggest that identification of patients with underweight BMI adds discriminatory power for identifying patients with pulmonary TB, and that routine BMI assessment should therefore be considered in settings where clinicians are often required to make decisions about empiric TB treatment for high-risk patients while awaiting bacteriologic data. Although the clinical utility of any specific algorithm would require further validation, these findings suggest that consideration of BMI could add diagnostic value above and beyond symptom count alone.**”(Lines 336 – 342)**

Query 10: It is not clear if the authors followed the STROBE guidelines when writing this manuscript? They would greatly help improve it.

**Response: We have reviewed the STROBE guidelines for case/control studies during the revision process. The Manuscript has been revised to clearly indicate the study design and setting, participants, data collection process, variables (outcome and predictors). We have included how we dealt with missing data as well as a description of the participant characteristics following STROBE guidelines.**
